# Editorial Note: Pathogenic *KRAS* variants disrupt structure and dynamics: Insights from integrated computational analyses

**DOI:** 10.1371/journal.pone.0355660

**Published:** 2026-08-13

**Authors:** The PLOS One Editors

The *PLOS One* Editors issue this Editorial Note to inform readers of the following issues that were noted after this article’s [1] publication:

- The articles cited as References 6, 7, 22, 23, 60, and 61 do not appear to support the respective corresponding cited statements.
- The article cited as Reference 65 was retracted after [1] was submitted but prior to its acceptance. PLOS considers that this reference is not crucial in supporting the research or conclusions reported in [1], and its corresponding cited statement remains supported by other references.

Corresponding author AU acknowledged the concerns and noted that the article cited as Reference 17 also does not appear to support its corresponding cited statement; however, the Editors consider that the cited statement remains supported by other references.

For the other references identified as not supportive of their corresponding cited statements, the author provided the following alternative references as replacements:

- The article originally cited as Reference 6 is replaced by the article cited here as [2];
- The article originally cited as Reference 7 is replaced by the article cited here as [3];
- The article originally cited as Reference 22 is replaced by the article cited here as [4];
- The article originally cited as Reference 23 is replaced by the article cited here as [5];
- The article originally cited as Reference 60 is replaced by the article cited here as [6];
- The article originally cited as Reference 61 is replaced by the article cited here as [7].
